# Balsam-Pear-Skin-Like-Structure Polyvinylidene Fluoride/Ethylene–Vinyl Alcohol Fibrous Membrane for Highly Efficient Oil/Water Separation Through One-Step Electrospinning

**DOI:** 10.3390/polym17101389

**Published:** 2025-05-18

**Authors:** Qijiao Jiang, Jinpeng Mo, Shaobo Han, Xi Liu, Baoliu Qu, Juan Xie, Xianfeng Wang, Jing Zhao

**Affiliations:** College of Textile Science and Engineering, Wuyi University, Jiangmen 529020, China

**Keywords:** electrospinning, balsam-pear-skin-like structure, water/oil separation, phase separation

## Abstract

The rapid growth of industrial activities has significantly increased oil demand, leading to wastewater contamination with oil and causing severe environmental pollution. Traditional oil–water separation techniques, such as gravity separation, filtration, and chemical treatments, are hindered by low efficiency, high energy consumption, and secondary pollution. Membrane separation technology has emerged as a promising solution due to its simplicity, low energy consumption, and high efficiency. In this study, we report the fabrication of a novel polyvinylidene fluoride/ethylene–vinyl alcohol (PVDF/EVOH) nanofibrous membrane (NFM) with a unique balsam-pear-skin-like structure using a one-step electrospinning process. The membrane’s superhydrophobicity and superoleophilicity were achieved via water vapor-induced phase separation (WVIPS), by optimizing the rheological properties and mixing ratio of EVOH and PVDF precursor solutions. The resulting PVDF/EVOH (PE12-3) NFM exhibits exceptional properties, achieving separation efficiencies of 99.4% for heavy oil and 98.9% for light oil, with a heavy oil flux of 18,020 L m^−2^ h^−1^—significantly surpassing previously reported performances. Additionally, the membrane shows excellent recyclability, making it ideal for large-scale oil–water separation in wastewater treatment and environmental remediation. This one-step fabrication strategy offers an efficient and scalable approach for developing high-performance membranes to tackle oil pollution in water.

## 1. Introduction

The rapid expansion of industrial activities has led to a significant increase in the demand for oil resources. However, oil extraction, transportation, and storage processes inevitably generate wastewater containing oil, resulting in resource wastage and environmental pollution [1,2,3]. The growing environmental concerns related to industrial oil spills and wastewater contamination have driven extensive research into efficient oil–water separation technologies [4,5,6]. Traditional separation methods, such as gravity separation, filtration, and chemical treatment, are often limited by low efficiency, high energy consumption, and the potential for secondary pollution [7,8]. Therefore, there is an urgent need for efficient, cost-effective, and scalable technologies to remove oil pollutants from water.

Recently, membrane separation technology has gained increasing attention in the field of oil–water emulsion separation due to its simplicity, low energy consumption, and high separation efficiency [9,10,11]. Inspired by natural hydrophobic effects, such as those observed on lotus leaves and butterfly wings, superwetting membranes have emerged as promising candidates for oil–water emulsion separation. Researchers have explored materials with varying affinities for oil and water and developed rough surface structures to achieve superwetting properties for separation. Ma et al. reported a PI-based nanofibrous membrane modified with perfluorodecanethiol (PFDT) that exhibited ultrahigh flux and excellent reusability [12]. Yang et al. designed a PVDF–SiO_2_ fibrous membrane that efficiently separated oily wastewater using a one-step electrospinning process [13]. Cheng et al. developed a Janus membrane with asymmetric wettability, demonstrating excellent recyclability and chemical resistance [14]. Despite these advances, challenges remain in achieving membranes with controlled pore structures, tunable surface roughness, and scalable fabrication processes, especially for the efficient treatment of stabilized oil–water emulsions. Electrospinning, a versatile technique for fabricating nanofibrous membranes (NFMs), offers advantages such as high surface area, tunable porosity, and controllable fiber morphology [15,16,17,18,19]. However, precise control over fiber surface topology and membrane wettability, especially through scalable and eco-friendly methods, remains underexplored.

In this study, we prepared a superwetting NFM featuring a rough, balsam-pear-skin-like structure to enhance hydrophobicity using a facile one-step electrospinning technique. By adjusting the rheological properties and mixing ratio of ethylene–vinyl alcohol (EVOH) and polyvinylidene fluoride (PVDF) precursor solutions, we controlled water vapor-induced phase separation (WVIPS) and tailored the roughness and porosity of the NFMs. The morphology, chemical composition, and separation performance of the membranes at varying EVOH concentrations were systematically investigated. The resulting PVDF/EVOH NFMs demonstrated good oil adsorption capacities, high separation flux, and remarkable recyclability, making them promising candidates for large-scale oil–water separation applications.

## 2. Materials and Methods

### 2.1. Materials

PVDF (Mw = 320,000) was purchased from Decheng Chemical Co., Ltd., Jiangmen, China. EVOH (density = 1.2 g/cm^3^, ethylene content = 44 mol%, Mw = 72,000 g/mol) was obtained from Kuraray Co., Ltd., Tokyo, Japan. N,N-dimethylacetamide (DMAc, ≥99.5%) was purchased from Thermo Fisher Scientific, Waltham, MA, USA. Reactive blue, petroleum ether (A.R.), isooctane (A.R.), cyclohexane (A.R.), dichloromethane (A.R.), chloroform (A.R.), and Sudan red II were all obtained from Macklin Biochemical Technology Co., Ltd., Shanghai, China.

### 2.2. Fabrication of PVDF/EVOH NFM

The pure PVDF solution was prepared by dissolving PVDF powder in DMAc solvent, achieving a PVDF concentration of 12 wt%. For the PVDF/EVOH solution, both PVDF powder and EVOH particles were dissolved in DMAc solvent. All polymer solutions were magnetically stirred at 85 °C for 12 h until complete dissolution. In this case, the PVDF concentration remained at 12 wt%, while the EVOH concentrations varied at 1, 2, 3, and 4 wt%, respectively. The uniform solution was then transferred into plastic syringes equipped for electrospinning, with a metal syringe needle connected to the positive electrode. The solution was extruded at a rate of 1.5 mL/h under an applied voltage of 30 kV. The electrospinning chamber was maintained at a temperature of 23–25 °C and a relative humidity of 85–95%. The resulting PVDF/EVOH fibers were collected on a greased paper substrate fixed to a rotating drum collector, operating at 50 rpm and positioned 22 cm from the needle tip. The pure PVDF fibers were labeled as PVDF NFM, and the composite PVDF/EVOH fibers were designated as PE12-1, PE12-2, PE12-3, and PE12-4 NFMs, respectively.

### 2.3. Characterization

The morphologies of the NFMs were examined using a scanning electron microscope (ZEISS GeminiSEM 300, Jena, Germany). To study the surface chemical structure, a Nicolet IS50 Fourier transform infrared spectrometer (Thermo Fisher, Waltham, MA, USA) was employed. FTIR analysis was performed in ATR mode. The surface chemical element composition of the NFMs was tested with an X-ray photoelectron spectrometer (XPS, Thermo Scientific K-Alpha, Thermo Escalab 250Xi, Waltham, MA, USA). The crystallinity of the NFMs was evaluated through X-ray diffraction (XRD, Rigaku Ultima IV, Tokyo, Japan). The surface roughness and profile measurements of the NFMs were conducted using an ultra-depth three-dimensional microscope (VHX-7000, KEYENCE, Osaka, Japan). The pore characteristics were investigated using a capillary flow porometer (CFP-1100AI, Ithaca, NY, USA) at a constant pressure of 40 psi. The porosity of the NFMs was determined using the same method as before [15,16]. The porosity of the membranes was then calculated as follows:

(1) $$Porosity=\frac{D_{0}-D_{1}}{D_{0}}\times 100\%$$

where $D_{0}$ and $D_{1}$ are the densities of the raw material and the fibrous membrane. The water contact angles (WCAs) on the NFMs were measured with a DSA100-Kruss contact angle goniometer (FSP GROUP, Hamburg, Germany). The absorbance of the samples was tested by UV-Vis-NIR photometer (UV-3600Plus, SHIMADZU, Kyoto, Japan).

### 2.4. Measurements

The air permeability of the NFMs was analyzed utilizing a YG461E-III automated air permeability tester (Ningbo Textile Instrument Factory, Ningbo, China). The water vapor transmission WVT (kg m^−2^ d^−1^) rate of the NFMs was evaluated with a YG216-II moisture permeability tester (Wenzhou Darong Textile Instrument Co., Ltd., Wenzhou, China), employing the inverted cup method at 38 °C and 50% relative humidity. The moisture permeabilities were calculated using the following equation:

(2) $$WVT\;\mathrm{rate}=\frac{m_{1}-m_{2}}{A}\times 24$$

where A (m^2^) stands for the test area, and $m_{1}-m_{2}$ (g) represents the mass of the decreased water. The flux and separation efficiency of the NFMs were tested using a custom apparatus, in which the PVDF/EVOH NFM was secured between two glass tubes. Oil–water mixtures were prepared by mixing 10 mL of dyed water with 90 mL of each respective solvent: petroleum ether, isooctane, cyclohexane, dichloromethane, and chloroform. These mixtures were then poured onto the NFM to enable separation. As the oil permeated the NFM, the volume of the remaining water was promptly recorded. The NFM was rinsed with alcohol following each separation, and this procedure was carried out for ten cycles. The oil flux J (L m^−2^ h^−1^) was determined according to the following formula:

(3) $$J=\frac{V}{S\times t}$$

in which t (h) is the time and S (m^2^) is the test area of the NFM. The separation efficiency η (%) was computed using the equation:

(4) $$\eta =\frac{V}{90}\times 100\%$$

The value of 90 mL corresponds to the initial volume of the oil phase (petroleum ether, isooctane, etc.), in which V(L) represents the volume of collected oil. A total of 0.5 g of petroleum ether and 0.05 g of Span 80 was added to a flask, followed by 49.5 g of deionized water. After vigorous stirring for 5 min, a petroleum ether/water emulsion formed. Then, 0.01 g of the fibrous membrane sample was placed into a beaker containing 20 g of the prepared emulsion. The sample was allowed to absorb oil droplets in the emulsion for 2 days. A optical microscope (DM2700M; Leica Microsystems GmbH, Wetzlar, Germany) was used to observe the emulsion before and after oil absorption, and a UV-Vis-NIR spectrophotometer (UV-3600Plus, SHIMADZU, Kyoto, Japan was employed to analyze the absorbance of the emulsion in the visible light region before and after oil absorption. All key performance tests, including oil–water separation efficiency, flux, contact angle, and adsorption capacity, were conducted with independent replicates (n ≥ 5) to minimize experimental errors. All quantitative results are presented as mean ± standard deviation.

## 3. Results

### 3.1. Construction of Balsam-Pear-Skin-like-Structure PVDF/EVOH NFM

**Figure 1:**
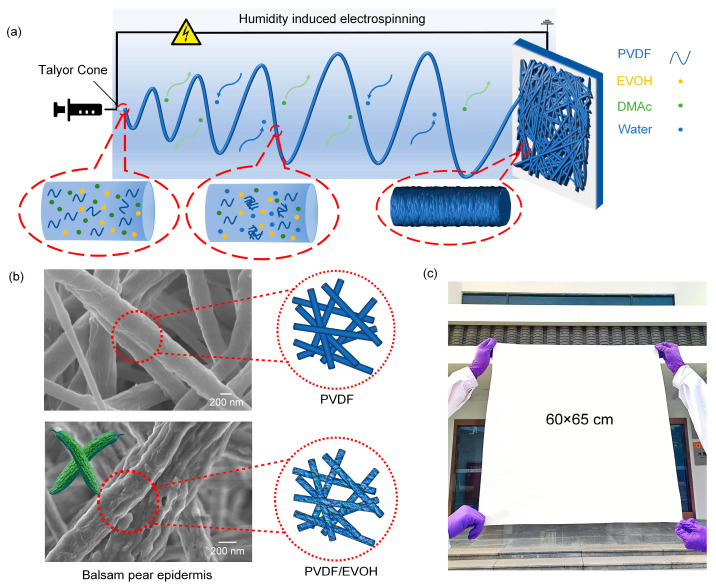
(**a**) Schematic illustration of the electrospinning process for PVDF/EVOH composite NFMs under high-humidity conditions. (**b**) SEM images showing the smooth surface structure of pure PVDF fibers and the balsam-pear-skin-like structure of PVDF/EVOH fibers. (**c**) Photograph of a large-scale PVDF/EVOH NFM.

In this study, we prepared a PVDF/EVOH composite NFM with a balsam-pear-skin-like structure for oil/water separation using a facile, one-step electrospinning technique, as illustrated in Figure 1a. In this process, a charged polymer solution was ejected from the needle tip, where it initially formed a Taylor cone under the applied electric field. As the jet traveled through the electric field, solvent volatilization was enhanced, and water vapor—acting as a nonsolvent—exchanged with the solvent in the polymer solution. This exchange accelerated solvent evaporation and the diffusion of nonsolvent molecules, leading to phase separation and the formation of fibrous structures with protrusions and depressions [20]. As a result, PVDF/EVOH fibers with a balsam-pear-skin-like structure were developed via water vapor-induced phase separation (WVIPS) (Figure 1b). The incorporation of hydrophilic EVOH accelerated this process [21]. Scanning electron microscope (SEM) images of the pure PVDF fibers revealed a relatively smooth surface with a uniform fiber diameter distribution, which confirmed the critical role of EVOH in the creation of epidermis-like structures. Due to the straightforward fabrication process, large-sized PVDF/EVOH NFMs (60 × 65 cm) can be easily produced (Figure 1c).

### 3.2. Morphology and Microstructure of PVDF/EVOH NFM

**Figure 2:**
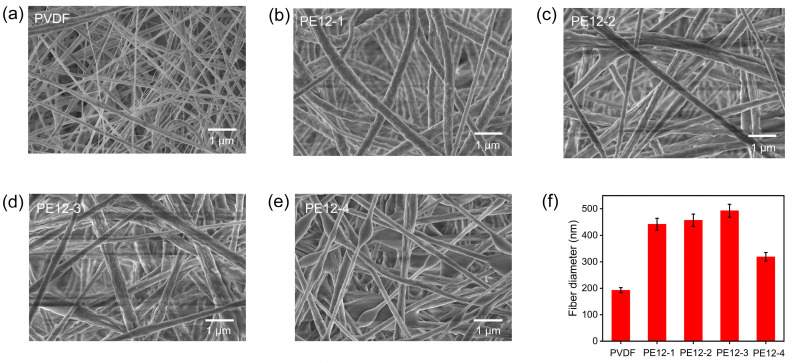
SEM images of (**a**) PVDF and (**b**–**e**) PVDF/EVOH composite NFMs. (**f**) Fiber diameter distribution for PVDF and PVDF/EVOH composite NFMs.

To investigate the effect of varying EVOH concentrations on the morphologies of PVDF/EVOH composite NFMs, EVOH concentrations were varied at 1, 2, 3, and 4 wt%. The morphological images of the PVDF and PVDF/EVOH fibers are shown in Figure 2a–e. The pure PVDF fibers exhibited a random orientation and a relatively smooth surface with an average fiber diameter of 193 nm (Figure 2a). As the EVOH concentration increased, the morphology of the fibers changed. At 1 wt% EVOH (Figure 2b), the fibers displayed a more distinct rough surface compared to pure PVDF fibers, and the average fiber diameter increased to 442 nm. As EVOH concentration increased to 3 wt%, the average fiber diameter gradually increased to 493 nm (Figure 2c,d), and the unique balsam-pear-skin-like structure formed on the surface of the fibers. The formation of thicker fibers was due to the increased concentration of the polymer solution. However, at a higher EVOH concentration of 4 wt% (Figure 2e), a reduction in the average fiber diameter to 319 nm was observed (Figure S1), along with the emergence of bead-like structures. This decrease in fiber diameter and the formation of beads are likely due to the lower viscosity of the spinning solution at this higher EVOH content (Figure S2), which leads to insufficient fiber elongation and the formation of beads during electrospinning [22,23].

### 3.3. Surface Chemical and Physical Properties of PVDF/EVOH NFMs

**Figure 3:**
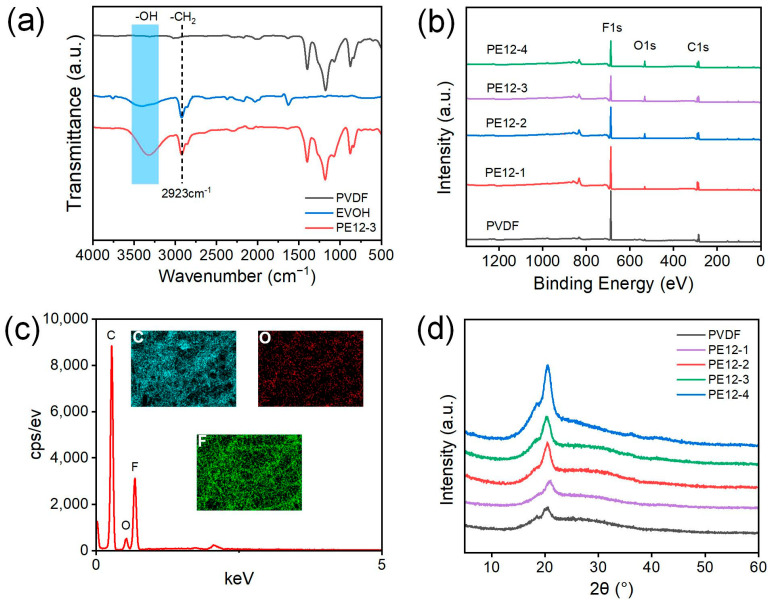
(**a**) FTIR spectra of PVDF and EVOH and PE12-3 NFMs. (**b**) XPS spectra of the PVDF and different PVDF/EVOH NFMs. (**c**) The EDS and elemental mapping images of C, O, and F for the PE12-3 NFM. (**d**) XRD curves of PVDF NFM sample and different PVDF/EVOH NFMs samples.

Figure 3a shows the Fourier transform infrared (FTIR) spectra of pure PVDF NFM and EVOH and PVDF/EVOH NFMs. Compared to the pure PVDF NFM, the PE12-3 NFM exhibited a stretching vibration peak of −CH_2_ at 2923 cm^−1^, which was attributed to the asymmetric −CH_2_ stretching vibration of the EVOH component [24]. A broad peak between 3200 and 3500 cm^−1^ was also observed, corresponding to the stretching vibration of −OH groups [25,26]. These results indicate that EVOH is indeed present in the PE12-3 NFMs. To further analyze the chemical composition on the surface of the NFMs, X-ray photoelectron spectroscopy (XPS) was used to study the surface of PVDF/EVOH membranes with varying EVOH concentrations, as shown in Figure 3b. A new peak for the O element appeared in all PVDF/EVOH membranes, and its intensity increased with the EVOH concentration. The detailed elemental compositions of F, C, and O in the PVDF/EVOH NFMs are listed in Table S1. As the EVOH content increased, the F element content decreased from 53.36% to 37.57%, the C element content increased from 46.42% to 52.89%, and the O element content gradually rose to 9.55%. These findings confirm the incorporation of EVOH into the PVDF NFMs.

The energy dispersive X-ray spectroscopy (EDS) spectrum confirmed the presence of EVOH on the external surface of the composite membrane, with a peak attributed to oxygen atoms appearing due to the addition of EVOH (Figure 3c). The crystalline phases of PVDF and PVDF/EVOH NFMs were analyzed using X-ray diffraction (XRD), as shown in Figure 3d. Diffraction peaks for pure PVDF NFM were observed at 2θ = 18.5°, 20.4°, and 26.6°, corresponding to the α (010), α (110), and α (021) planes, respectively, characteristic of the α-phase crystal structure [27]. Notably, the diffraction pattern of PVDF/EVOH NFMs differed from that of pure PVDF, due to the incorporation of EVOH. A significant change in the crystalline structure was observed for the membrane with 4 wt% EVOH, which can be attributed to the hydroxyl groups of EVOH-facilitated PVDF crystallization, consistent with findings reported in the literature [28].

### 3.4. Porous Structure of PVDF/EVOH NFMs

**Figure 4:**
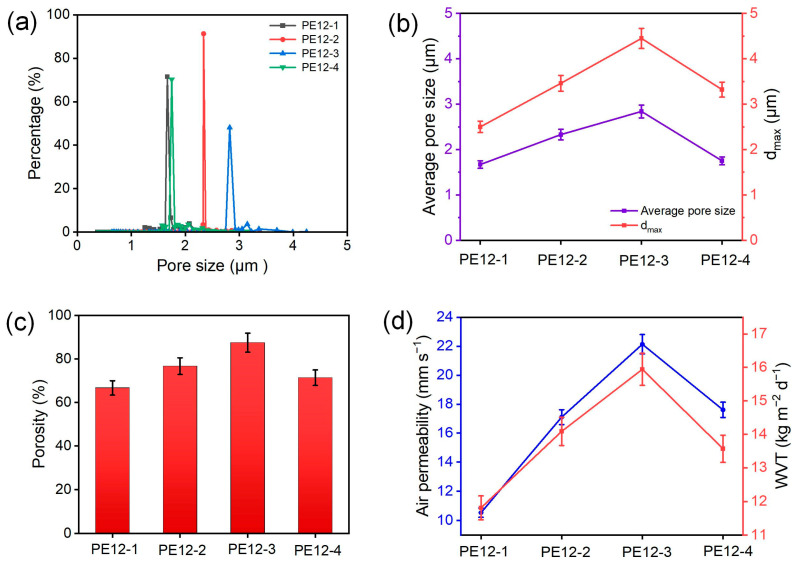
(**a**) Pore size distribution, (**b**) average pore size and dmax, (**c**) porosity, and (**d**) air permeability and WVT rate of different PVDF/EVOH NFMs.

Figure 4 displays the pore characteristics of different PVDF/EVOH NFMs. The pore size distribution of the PVDF/EVOH fiber membrane is relatively uniform, as shown in Figure 4a,b. When the EVOH concentrations were 1, 2, 3, and 4 wt%, the average pore sizes were 1.67, 2.33, 2.84, and 1.75 μm, respectively, while the maximum pore sizes (dmax) were 2.5, 3.46, 4.45, and 3.32 μm, respectively. When the EVOH concentration was 3 wt%, both the average pore size and dmax reached their peak values. This was because, at this concentration, the fibers had the largest average diameter, and the pores formed by these fibers stacking were larger. With EVOH concentrations of 1, 2, 3, and 4 wt%, the porosities of PVDF/EVOH NFMs were 66.7, 76.7, 87.5, and 71.4%, respectively, which was closely associated with the change in fiber diameters (Figure 4c). As the EVOH concentration increased from 1 to 3 wt%, the air permeabilities of the NFMs increased from 10.5 to 22.1 mm/s (Figure 4d). However, when the EVOH concentration was further increased to 4 wt%, the air permeability decreased to 17.6 mm/s, which was directly related to the changes in pore size. Moreover, the water vapor transmission (WVT) rate shows a positive correlation with porosity (PE12-3 with 87.5% porosity exhibits a WVT rate of 15.94 kg m^−2^ d^−1^). When the EVOH content increased to 4 wt%, the reduced porosity led to a decrease in the WVT rate to 13.57 kg m^−2^ d^−1^. These results validate the influence of membrane structure on mass transfer performance.

### 3.5. Surface Wettability of PVDF/EVOH NFMs

**Figure 5:**
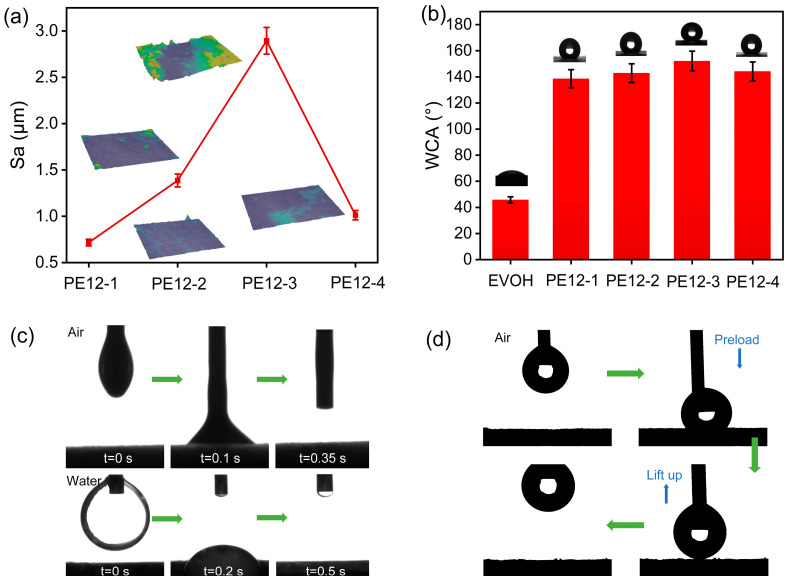
(**a**) The Sa values of different PVDF/EVOH NFMs; the inset is the corresponding optical profilometry images. (**b**) WCA of EVOH and different PVDF/EVOH NFMs and the corresponding optical profilometry images. (**c**) Rapid wetting diagram of an oil droplet in air and water. (**d**) Diagram of the process of a water droplet contacting, depressing, and detaching from the PVDF/EVOH NFM.

The surface roughness of the PVDF/EVOH NFMs were further investigated. Figure 5a displays the corresponding Ra values of the membranes detected via a noncontact optical profilometry analysis method. As EVOH concentrations increased from 1 to 3 wt%, the Sa values increased from 0.71 to 2.89 μm, which may be related to the increased concave–convex structures on the fibers’ surfaces. When the concentration of EVOH was further increased to 4 wt%, the Sa value of the NFM decreased to 1.0 μm, which may be associated with the bead-like structures on the fibers. Subsequently, we studied the water contact angle (WCA) of the PVDF/EVOH NFMs with different EVOH concentrations. The WCAs of the PE12-1, PE12-2, PE12-3, and PE12-4 NFMs were 138.6°, 142.9°, 152.1°, and 144.2°, respectively, which is consistent with the changes in surface roughness (Figure 5b). The PE12-3 NFM was chosen to analyze oleophilicity, as shown in Figure 5c. The membrane exhibited remarkable superoleophilicity, rapidly wetting oil droplets, whether in air or water. In practical applications, where the oil phase serves as the continuous phase, we further evaluated the oil–water adhesion of the PE12-3 NFM (Figure 5d). When water droplets made contact with the membrane surface and were compressed, their shape remained unchanged after being quickly lifted, demonstrating the membrane’s low water adhesion.

### 3.6. Oil Absorption Capability of PVDF/EVOH NFMs

**Figure 6:**
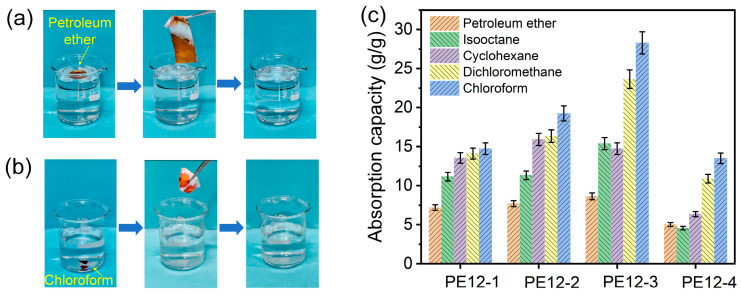
Photographs showing the selectively adsorbed (**a**) petroleum ether and (**b**) chloroform. (**c**) Comparison of absorption capacity of different PVDF/EVOH NFMs for five types of oils.

Thanks to their combined hydrophobicity and superoleophilicity, the PVDF/EVOH NFMs hold significant potential as an adsorbent material for oil removal from water. As exhibited in Figure 6a, a droplet of petroleum ether was gently added to the surface of the water in a glass, creating an oil layer. Next, a section of the fabricated PE12-3 NFM was placed on top of the oil layer. The petroleum ether was completely absorbed within seconds, leaving no visible oil on the surface of water. In addition, liquids with a density greater than water, such as chloroform, were used to assess the absorption capacity of the PE12-3 NFM. As the hydrophobic membrane was submerged in water near chloroform, it quickly absorbed the chloroform due to its strong superoleophilicity (Figure 6b).

The adsorption capacities of the PVDF/EVOH NFMs for different oils were also tested, as demonstrated in Figure 6c. As part of an absorption test, the membrane was immersed in the oil, then quickly removed after it reached saturation and weighed promptly to prevent evaporation. The test was repeated three times for each oil. After each measurement, the membrane was rinsed with ethanol to eliminate the absorbed oil and subsequently dried at 60 °C. The results showed that PE12-3 sample achieved the highest oil absorption, with a weight increase of up to 28.29 g/g. Its excellent adsorption performance can be attributed to its large surface roughness and highest porosity, which provide a larger adsorption area and more oil transport channels. The weight increase varied for different oils, with oil density and viscosity being two key characteristics influencing the experimental results. Taking PE12-3 as an example, the membrane showed a significantly higher adsorption capacity for high-density oils compared to low-density oils. For instance, when adsorbing chloroform (density 1.49 g/cm^3^), the membrane’s weight increase was 28.29 g/g, while for petroleum ether (density 0.63 g/cm^3^), the weight increase was only 8.64 g/g. Additionally, oil viscosity affects the adsorption capacity. High-viscosity oils, such as petroleum ether, have poor fluidity, which may lead to a reduced adsorption capacity [29]. In contrast, low-viscosity oils, such as chloroform, have better fluidity, allowing them to penetrate the material’s pores more quickly, thereby increasing the adsorption capacity [30]. To evaluate the oil absorption performance of the PE12-3 sample, we compared it with other PVDF-based oil-absorbing materials reported in the literature, as shown in Table S2 [31,32,33,34]. The PVDF/EVOH NFM exhibited good oil absorption capacity, with a range of 8.64–28.29 g/g, significantly higher than that of PVDF aerogel (3.10–6.78 g/g) and EG-PVDF foam (4.1–7.0 g/g). Furthermore, compared to PVDF/CoFe_2_O_4_ fibrous composite (18.074 g/g) and PVDF/PMMA open-cell foams (9.4–26 g/g), the PE12-3 sample demonstrated superior oil absorption performance for various oils, such as petroleum ether, isooctane, cyclohexane, and dichloromethane.

### 3.7. Oil/Water Separation of PVDF/EVOH NFMs

**Figure 7:**
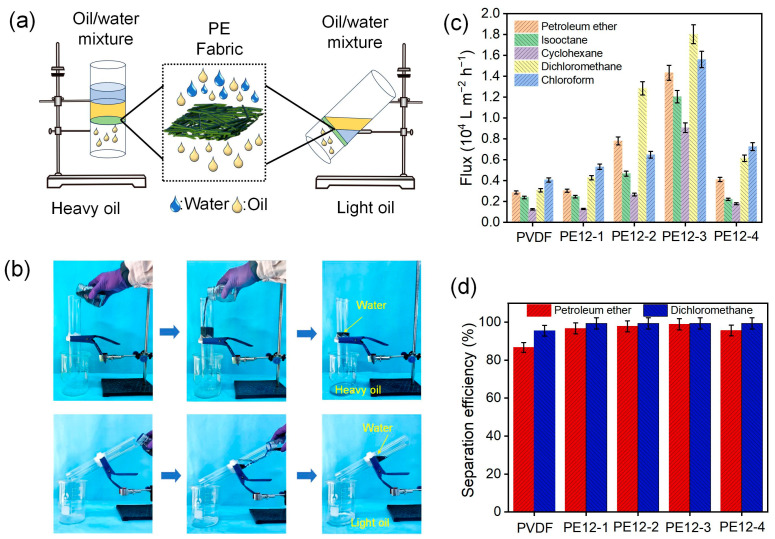
(**a**) Diagram illustrating the separation procedure of PVDF/EVOH NFM for heavy and light oils. (**b**) Oil–water separation procedure of heavy oil (methylene chloride) and light oil (petroleum ether) and water (dyed with active blue). (**c**) Separation flux of PVDF/EVOH NFMs with different types of oil. (**d**) Separation efficiency of PVDF/EVOH NFMs with petroleum ether and dichloromethane.

Figure 7a depicts a schematic of the separation process for a mixed solution of heavy and light oils. To separate the heavy oil mixture, the device was positioned vertically, while the light oil mixture was separated with the device inclined (Figure 7b). In order to investigate the separation flux and efficacy of the PVDF/EVOH NFMs, experiments were carried out using 90 mL of solvent mixed with 10 mL of dyed water. The PVDF/EVOH NFMs were used to separate a range of oil/water mixtures, including petroleum ether/water, isooctane/water, cyclohexane/water, dichloromethane/water, and chloroform/water.

As shown in Figure 7c, the separation flux of both heavy and light oils increased as the EVOH concentration rose, suggesting a positive correlation between the average pore size, porosity, hydrophobicity, and the oil-separation flux. The maximum flux of PE12-3 NFM reached 18,020 L·m^−2^·h^−1^ for the separation of heavy oil (dichloromethane/water mixture) and 14,331 L·m^−2^·h^−1^ for the separation of light oil (petroleum ether/water mixture). Figure 7d demonstrates the separation efficiencies of PVDF/EVOH NFMs for petroleum ether and dichloromethane. It can be found that the highest separation efficiency of PE12-3 NFM for dichloromethane/water was 99.4% and, for petroleum ether/water, 98.9%. The ability of the PVDF/EVOH NFMs to handle various oil–water mixtures is influenced by the viscosity and density of the oil [35,36]. These findings highlight the membrane’s versatility in processing different oil–water mixtures, as reflected by the varying fluxes and separation efficiencies.

**Figure 8:**
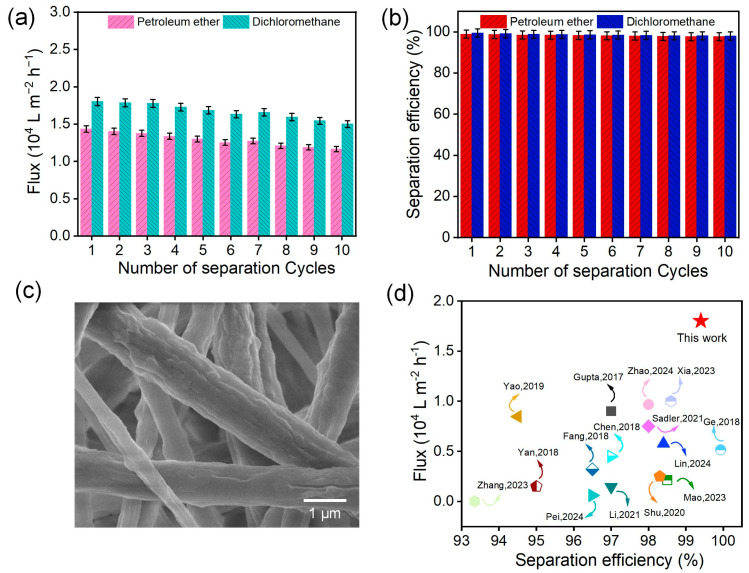
(**a**) Separation flux and (**b**) separation efficiency of the PE12-3 sample over multiple separation cycles. (**c**) SEM image of the PE12-3 sample’s surface after multiple separation cycles. (**d**) Performance comparison of PE12-3 NFM with other previously reported materials [8,37,38,39,40,41,42,43,44,45,46,47,48,49,50].

The stability of hydrophobicity and superoleophilicity in practical applications is crucial. The balsam-pear-skin-like structure PE12-3 NFM demonstrated excellent recycling performance, maintaining consistent flux and separation efficiency (Figure 8a,b) with only minimal fluctuations, even after 10 cycles. SEM images (Figure 8c) of the membrane’s surface after multiple oil–water separation cycles showed no noticeable changes, confirming the material’s outstanding stability. Figure 8d presents a comparison of the separation efficiency and oil flux with those of other materials reported in previous studies. The PE12-3 NFM with a balsam-pear-skin-like structure exhibited exceptional oil–water separation efficiency and remarkable oil flux. The flux for heavy oil was two to four times greater than that of previously reported materials [8,37,38,39,40,41,42,43,44,45,46,47,48,49,50]. Therefore, the PE12-3 NFM demonstrated exceptional oil–water separation performance and recyclability, highlighting its potential for large-scale applications.

**Figure 9:**
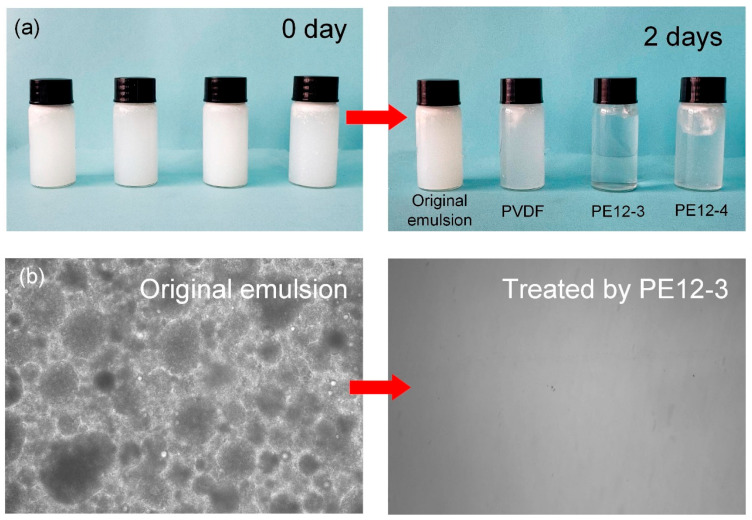
(**a**) Optical images of the PVDF, PE12-3, and PE12-4 samples before and after 2 days of emulsion absorption. (**b**) Optical micrographs of the PE12-3 sample before and after 2 days of emulsion absorption.

The underwater absorption behavior of NFM samples is directly related to their separation performance in oil–water emulsions. As shown in Figure 9a, the petroleum ether/water emulsion treated with the PE12-3 NFM exhibited a noticeable clarification within 2 days, indicating a high oil droplet adsorption capacity. In contrast, emulsions treated with the PVDF membrane and other PE membranes remained semi-transparent, suggesting the presence of unadsorbed residual oil droplets. Optical microscopy observations of the emulsions before and after adsorption (Figure 9b) revealed that the original emulsion contained uniformly dispersed spherical oil droplets. However, after treatment with the PE12-3 membrane, no significant oil droplets were detected in the liquid phase. This contrast can be attributed to the unique surface characteristics of the PE12-3 membrane: its balsam-pear-skin-like rough structure (Sa = 2.89 μm) and high porosity (87.5%) work synergistically to enable rapid oil droplet capture through capillary action and surface energy differences. In comparison, the smooth surface of the PVDF membrane (Sa = 0.71 μm) and the bead-like defect structure of the PE12-4 membrane (Figure 2e) significantly weakened their adsorption kinetics.

**Figure 10:**
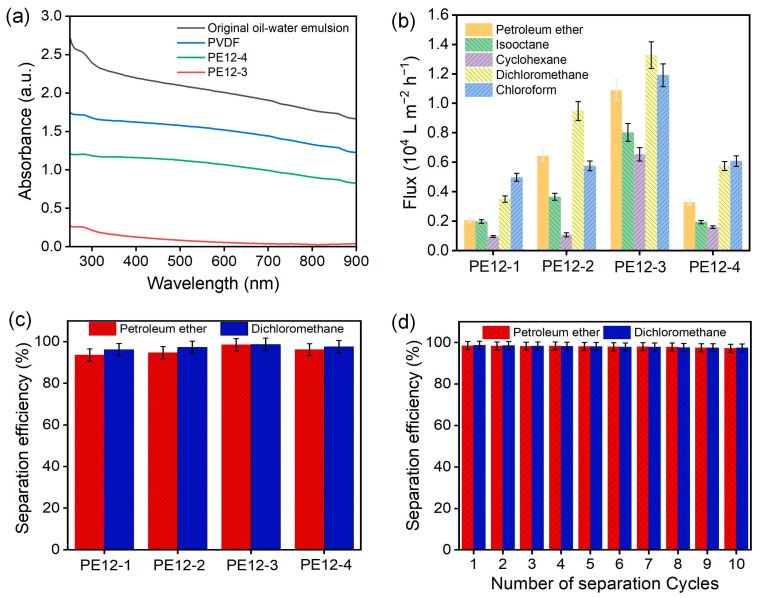
(**a**) UV-visible spectral analysis of emulsions before and after absorption by PVDF, PE12-3, and PE12-4 samples. (**b**) Separation flux of PVDF/EVOH NFMs for various oil–water emulsions. (**c**) Separation efficiency of PVDF/EVOH NFMs for petroleum ether/water and dichloromethane/water emulsions. (**d**) Separation efficiency of the PE12-3 NFM for petroleum ether/water and dichloromethane/water emulsions over multiple separation cycles.

Furthermore, visible light spectral analysis of the emulsions before and after adsorption (Figure 10a) yielded similar conclusions. The original emulsion exhibited a visible light absorbance significantly higher than 1.6, indicating high turbidity. After treatment with the PE12-3 NFM, the absorbance dropped to near-baseline levels, confirming the efficient removal of oil droplets. In contrast, emulsions treated with the PVDF and PE12-4 membranes maintained absorbance values above 0.8, suggesting the presence of residual oil droplets that caused light scattering. These findings are in strong agreement with the optical microscopy observations (Figure 9b) and the clarification phenomenon seen in Figure 9a. Figure 10b presents the separation fluxes of PVDF/EVOH NFMs for various oil-in-water emulsions. As shown, the separation fluxes for both heavy oil/water and light oil/water emulsions initially increased and then decreased with rising EVOH concentration. The PE12-3 membrane exhibited the highest flux of 13,265 L·m^−2^·h^−1^ for the heavy oil/water emulsion and 10,891 L·m^−2^·h^−1^ for the light oil/water emulsion. Figure 10c displays the separation efficiencies of PVDF/EVOH membranes for petroleum ether/water and dichloromethane/water emulsions. The PE12-3 NFM demonstrated excellent performance, achieving separation efficiencies of 98.7% for dichloromethane/water and 98.5% for petroleum ether/water—values comparable to those achieved for pure oils—highlighting the membrane’s exceptional capability in emulsion treatment. Additionally, the PE12-3 NFM showed outstanding recyclability in emulsion separation. Even after 10 separation cycles, the efficiency remained consistently above 97.4% with minimal fluctuation (Figure 10d), confirming the excellent stability of the material.

## 4. Conclusions

In this study, we successfully fabricated PVDF/EVOH NFMs with a unique balsam-pear-skin-like structure using a straightforward one-step electrospinning technique. The incorporation of EVOH was pivotal in inducing phase separation during electrospinning, resulting in a rough, porous membrane with significantly enhanced oil–water separation performance. The optimal PE12-3 NFM demonstrated outstanding separation efficiencies of 99.4% for heavy oil and 98.9% for light oil. Notably, the heavy oil flux of the PE12-3 NFM reached 18,020 L m^−2^ h^−1^, surpassing the performance reported in previous studies by a considerable margin. For the separation performance of oil/water emulsions, the PE12-3 NFM exhibited excellent separation efficiencies of 97.4% for heavy oil/water emulsions and 97.2% for light oil/water emulsions. The maximum separation flux for the heavy oil/water emulsion reached 13,265 L·m^−2^·h^−1^. Furthermore, the membrane exhibited exceptional durability, retaining its structural integrity and high separation performance even after multiple cycles, demonstrating its recyclability. These results underscore the potential of balsam-pear-skin-like-structure PVDF/EVOH NFMs as an efficient, sustainable solution for oil–water separation, with promising applications in wastewater treatment, environmental remediation, and industrial oil recovery.

## Data Availability

The original contributions presented in this study are included in the article/Supplementary Material. Further inquiries can be directed to the corresponding authors.

## Supplementary Materials

The following supporting information can be downloaded at: https://www.mdpi.com/article/10.3390/polym17101389/s1, Figure S1: The fiber diameter distribution curves of PVDF and different PVDF/EVOH NFMs; Figure S2: The viscosities of the spinning solutions corresponding to PVDF and different PVDF/EVOH NFMs; Table S1: XPS data of PVDF and different PVDF/EVOH NFMs; Table S2: Comparison of oil absorption capacities of various PVDF-based oil-absorbing materials. References [31,32,33,34] are cited in the Supplementary Materials.

## Abbreviations

The following abbreviations are used in this manuscript:

NFM	Nanofibrous membrane
WVIPS	Water vapor-induced phase separation
PE	PVDF/EVOH
EVOH	Ethylene–vinyl alcohol
PVDF	Polyvinylidene fluoride
DMAc	N,N-dimethylacetamide
FTIR	Fourier transform infrared spectra
XPS	X-ray photoelectron spectroscopy
EDS	Energy dispersive X-ray spectroscopy
XRD	X-ray diffraction
WVT	Water vapor transmission
WCA	Water contact angle
