# Supplementary material for: Balsam-Pear-Skin-Like-Structure Polyvinylidene Fluoride/Ethylene–Vinyl Alcohol Fibrous Membrane for Highly Efficient Oil/Water Separation Through One-Step Electrospinning
Source: Polymers (Basel). 2025 May 18;17(10):1389. doi: 10.3390/polym17101389 (PMC12114631; doi:10.3390/polym17101389)
Supplement: Supplementary file 1 [file polymers-17-01389-s001.zip › polymers-3630042-supplementary.pdf]

Supporting Information for

Balsam-Pear-Skin-Like structure Polyvinylidene Fluoride/Ethylene-Vinyl Alcohol Fibrous Membrane for Highly Efficient Oil/Water Separation through One-Step Electrospinning

Qijiao Jiang, Jinpeng Mo, Shaobo Han, Xi Liu, Baoliu Qu, Juan Xie *, Xianfeng Wang and Jing Zhao *

College of Textile Science and Engineering, Wuyi University, Jiangmen 529020, China; 15603933290@163.com (Q.J.); mjp1830@163.com (J.M.); qdhanshaobo@126.com (S.H.); liuxi@wyu.edu.cn (X.L.); nwpuqubaoliu@163.com (B.Q.); wxf@dhu.edu.cn (X.W.)

* Correspondence: anna_jxie@163.com (J.X.); jingzhaoedu@126.com (J.Z.)

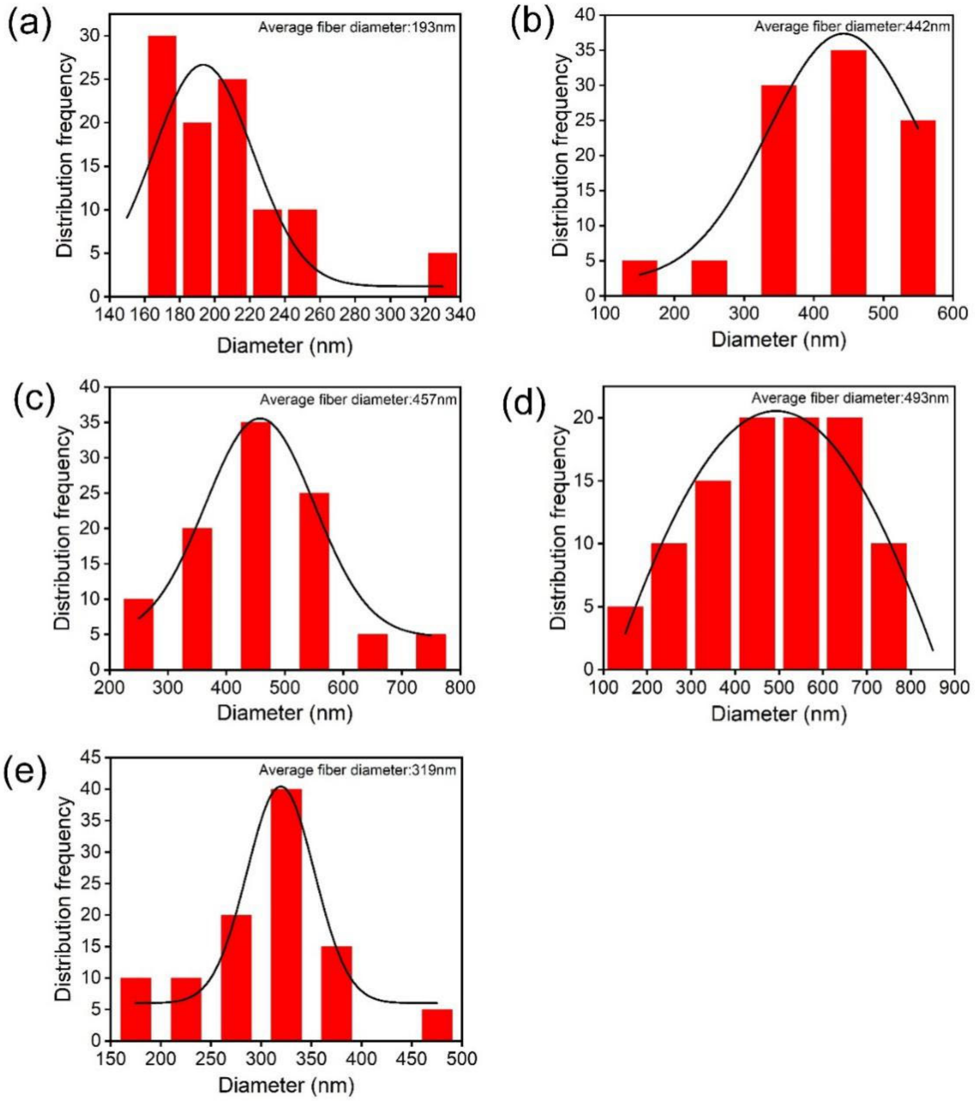

Figure S1. (a-e) The fiber diameter distribution curves of PVDF and different PVDF/EVOH NFMs.

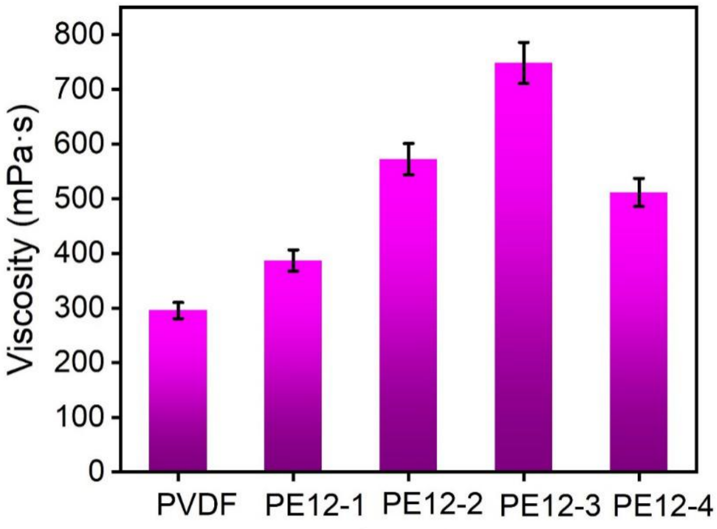

Figure S2. The viscosities of the spinning solutions corresponding to PVDF and different PVDF/EVOH NFMs.

Table S1. XPS data of PVDF and different PVDF/EVOH NFMs.

Sample	F%	O%	C%
PVDF	53.36	0.22	46.42
PE12-1	48.40	3.39	48.21
PE12-2	41.64	7.49	50.87
PE12-3	39.39	8.85	51.77
PE12-4	37.57	9.55	52.89

Table S2. Comparison of oil absorption capacities of various PVDF-based oil-absorbing materials.

Reference	Type of oil absorbent	Type of oil	Absorption capacity (g/g)
1	PVDF aerogel	hexane, toluene, gasoline, diesel, dichloromethane, chlorobenzene, cyclohexane	3.10-6.78
2	EG-PVDF foam	chloroform, soybean oil, engine oil, benzene, methanol, petroleum ether, n-hexane	4.1-7.0
3	PVDF/$CoFe_2O_4$ fibrous composite	motor oils	18.074
4	PVDF/PMMA open-cell foams	CCl_4, cyclohexane, diesel silicone oil, peanut oil	9.4-26
This research	PVDF/EVOH NFM	petroleum ether, isooctane, cyclohexane, dichloromethane, chloroform	8.64-28.29

References

(1) Chen, X.; Liang, Y. N.; Tang, X.-Z.; Shen, W.; Hu, X. Additive-free poly (vinylidene fluoride) aerogel for oil/water separation and rapid oil absorption. *Chem. Eng. J.* **2017,** *308*, 18-26.

(2) Tian, Y.; Ma, H. Solvent-free green preparation of reusable EG-PVDF foam for efficient oil-water separation. *Sep. Purif. Technol.* **2020,** *253*, 117506.

(3) Dorneanu, P. P.; Cojocaru, C.; Olaru, N.; Samoila, P.; Airinei, A.; Sacarescu, L. Electrospun PVDF fibers and a novel PVDF/$CoFe_2O_4$ fibrous composite as nanostructured sorbent materials for oil spill cleanup. *Appl. Surf. Sci.* **2017,** *424*, 389-396.

(4) Shi, Z.; Zhao, G.; Zhang, L.; Wang, G.; Chai, J. Ultralight and hydrophobic PVDF/PMMA open-cell foams with outstanding heat-insulation and oil-adsorption performances fabricated by CO_2 molten foaming. *J. CO_2 Util.* **2022,** *63*, 102108.
